# Unconventional Ductal Flow Restriction and Iatrogenic Pseudoaneurysm Plug Closure in Pulmonary Atresia With Intact Septum

**DOI:** 10.1016/j.jaccas.2025.106345

**Published:** 2025-12-03

**Authors:** John Wiegand, James Thompson

**Affiliations:** a Department of Pediatrics, University of South Florida, Tampa, Florida, USA; b Johns Hopkins All Children's Hospital, St Petersburg, Florida, USA

**Keywords:** cardiac catheterization, ductal flow restrictor, microvascular plug, pseudoaneurysm, pulmonary atresia

## Abstract

**Background:**

Pulmonary atresia with intact ventricular septum (PA/IVS) and right ventricular–dependent coronary circulation pose management challenges requiring creative transcatheter solutions when surgery is unfeasible.

**Case Summary:**

A small-for-gestational-age term neonate with PA/IVS and right ventricular–dependent coronary circulation developed hemodynamic instability after patent ductus arteriosus stenting, requiring deployment of a modified Medtronic Microvascular Plug within the patent ductus arteriosus stent to restrict pulmonary overcirculation. A left external iliac artery pseudoaneurysm was subsequently closed using a 4-mm Konar-A vascular plug.

**Conclusions:**

This case underscores the delicate balance between management of pulmonary over- and undercirculation in neonates with PA/IVS and highlights creative transcatheter strategies used to address these dual hemodynamic dilemmas. Early recognition of at-risk anatomy, and consideration for cardiac transplantation, are essential for survival in these high-risk patients.

**Take-Home Message:**

Unconventional transcatheter strategies may be essential in managing high-risk neonates with congenital heart disease.

## Visual Summary

**Figure:**
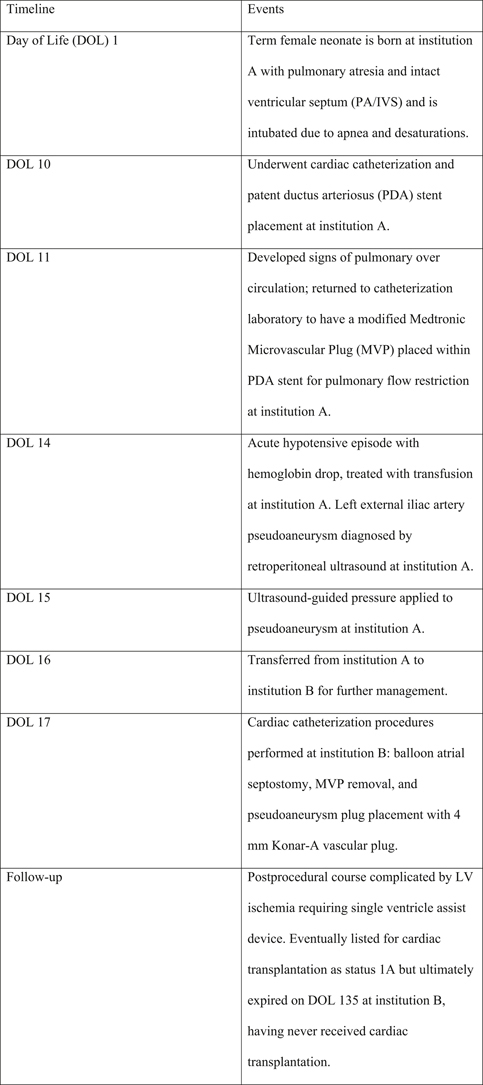

**Visual Summary:**
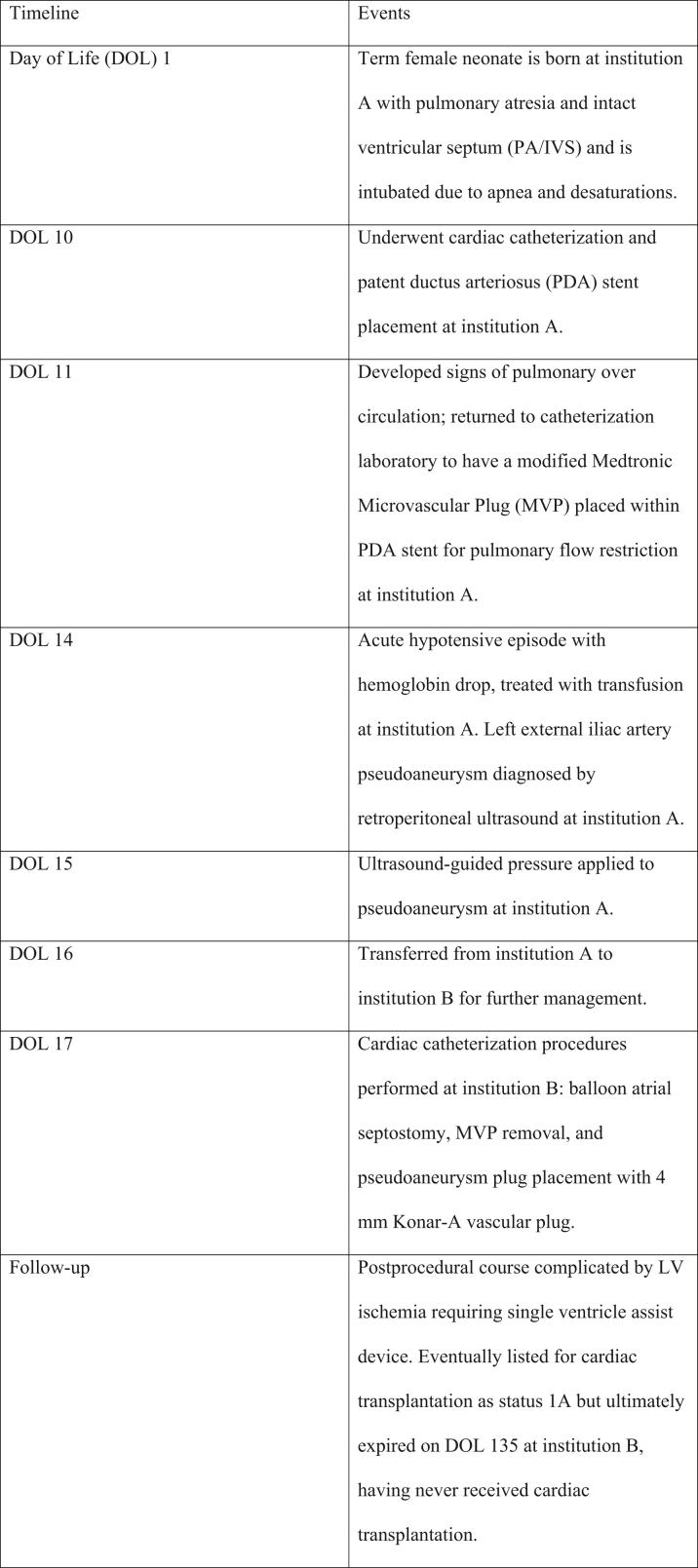
Timeline of Events LV = left ventricular.

## History of Presentation

**Figure 1:**
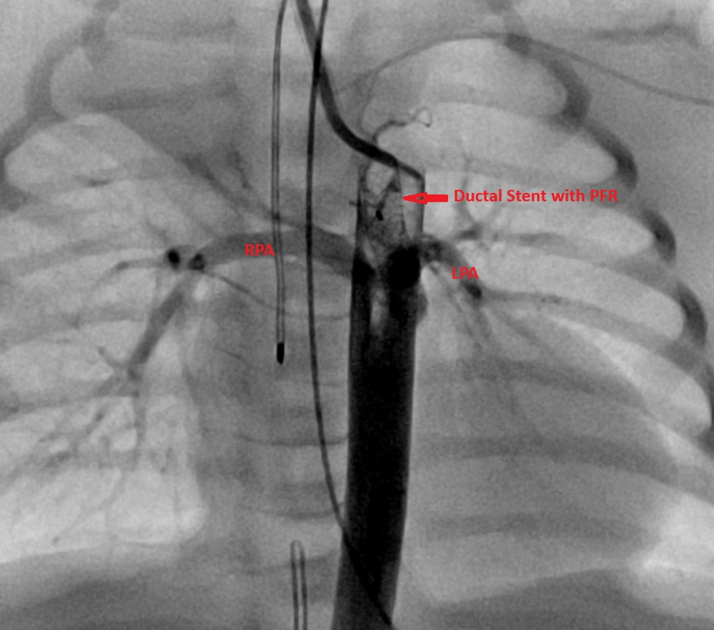
Anteroposterior View, Post-MVP Placement Anteroposterior angiographic view demonstrating the patent ductus arteriosus stent with the Medtronic microvascular plug (MVP) positioned within the ductal stent. The LPA and RPA are labeled for orientation. LPA = left pulmonary artery; PFR = pulmonary flow restrictor; RPA = right pulmonary artery.

**Figure 2:**
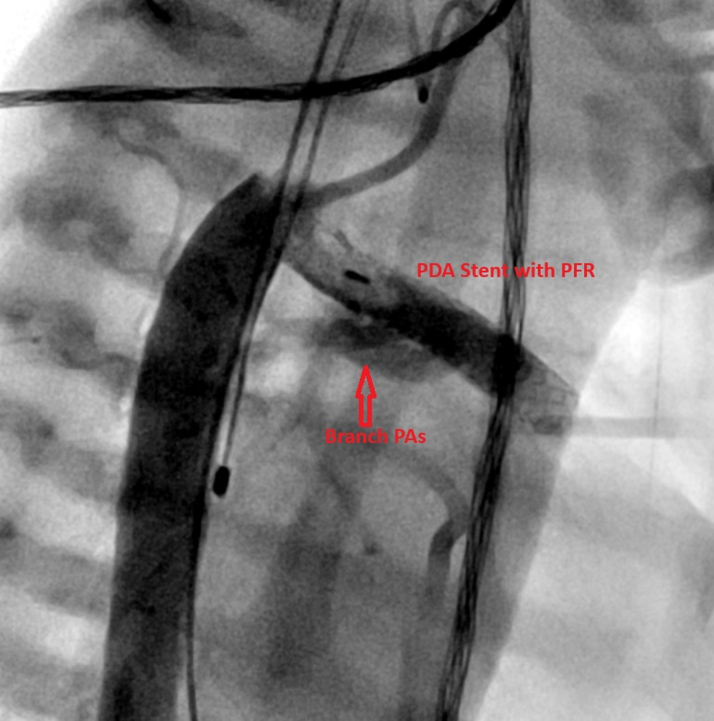
Lateral View, Post-MVP Placement Lateral angiographic view demonstrating the PDA with the Medtronic microvascular plug (MVP) positioned within the ductal stent. The right and left PAs are labeled as branch PAs for orientation. PA = pulmonary artery; PDA = patent ductus arteriosus; PFR = pulmonary flow restrictor.

A small-for-gestational-age female neonate born at 37 weeks and 2 days via spontaneous vaginal delivery (birth weight: 2.28 kg) was prenatally diagnosed with pulmonary atresia with intact ventricular septum (PA/IVS), confirmed postnatally. She was intubated for apnea and desaturations, extubated on day of life (DOL) 6, but reintubated on DOL 9 due to persistent left lung collapse not improved by aggressive chest physiotherapy before planned cardiac catheterization. Catheterization on DOL 10 confirmed right ventricular (RV)–dependent left coronary circulation. A 4- × 26-mm coronary stent was placed in the arterial duct to secure pulmonary flow. By DOL 11, she developed pulmonary overcirculation refractory to hypercarbia, with tachycardia, hypotension, and rising lactate (7-9 mmol/L), prompting urgent placement of a 5-mm modified Medtronic microvascular plug (MVP) within the ductal stent to restrict pulmonary flow. The MVP was fenestrated with two 3- to 4-mm openings created using electrocautery and deployed via the left femoral artery. Angiography obtained thereafter demonstrated adequate flow through the MVP with opacification of both pulmonary arteries (Figures 1 and 2, Videos 1 and 2).

A heparin infusion was started on DOL 12 for diminished pulses in the left lower extremity. On DOL 14, she developed a hematocrit drop (24.4%) and worsening lactate (12 mmol/L), which improved after blood transfusions. Retroperitoneal ultrasound revealed a left external iliac artery pseudoaneurysm with adjacent hemorrhage measuring 3.5 × 2.0 × 2.1 cm and extending into the left perinephric and paranephric spaces, and left femoral artery occlusion. Brain ultrasound was normal. Ultrasound-guided manual pressure was performed on DOL 15, and she was transferred to our institution on DOL 16 for further management.

On arrival, she remained intubated, sedated, and paralyzed with neuromuscular blockade, demonstrating oxygen saturations in the mid-70s. Auscultation revealed clear lungs and a continuous machine-like murmur in the second left infraclavicular space. The left foot was cool with delayed capillary refill (3 seconds); popliteal and femoral pulses were Doppler-detectable, but distal pulses (ie, dorsalis pedis, posterior tibial) were absent.

## Past Medical History

Aside from the aforementioned presentation, the patient otherwise had an uneventful pregnancy and delivery.

## Differential Diagnosis

At presentation, initial considerations included ductal-dependent lesions causing hypoxemia before echocardiographic confirmation of PA/IVS. Critical pulmonary stenosis may mimic PA/IVS but has some antegrade RV outflow. Tricuspid atresia causes cyanosis and reduces pulmonary flow but lacks atrioventricular continuity. Severe Ebstein anomaly can cause functional pulmonary atresia but was excluded by normal tricuspid leaflet morphology. Persistent pulmonary hypertension of the newborn was briefly considered but excluded by fixed outflow obstruction and ductal-dependent flow.

## Investigations

Echocardiography at birth demonstrated valvar PA/IVS, left coronary artery (LCA) atresia, severely hypoplastic and bipartite right ventricle with moderate-to-severe hypertrophy, severely hypoplastic tricuspid valve with minimal antegrade flow, a moderate-to-large patent ductus arteriosus (PDA) with left-to-right shunt and a peak gradient of 13 mm Hg, hypoplastic pulmonary arteries (3 mm each), small secundum atrial septal defect vs stretched patent foramen ovale with right-to-left shunt, and RV-dependent coronary circulation. The left ventricle and systemic outflow were normal. RV-to-coronary artery fistulae and a normally arising right coronary artery were visualized, but the LCA origin was not seen.

Computerized tomographic angiography on DOL 4 showed a 6- × 4.5-mm PDA with left-sided aortic arch and normal systemic anatomy. Catheterization on DOL 10 confirmed aortic origin of the right coronary artery, atretic LCA, and RV-to-coronary artery fistulae supplying the left coronary system. Repeat echocardiography at our institution on DOL 16 revealed a restrictive atrial septum and continuous left-to-right PDA shunting (peak gradient: 63 mm Hg). Repeat vascular ultrasound of the left lower extremity at our institution on DOL 16 showed evidence of a partially (20%) thrombosed and growing left external iliac artery pseudoaneurysm, measuring 4 × 3 × 3.3 cm, with a 2-mm neck with severe stenosis (peak velocity: 300 cm/s), and probable common femoral occlusion.

## Management

**Figure 3:**
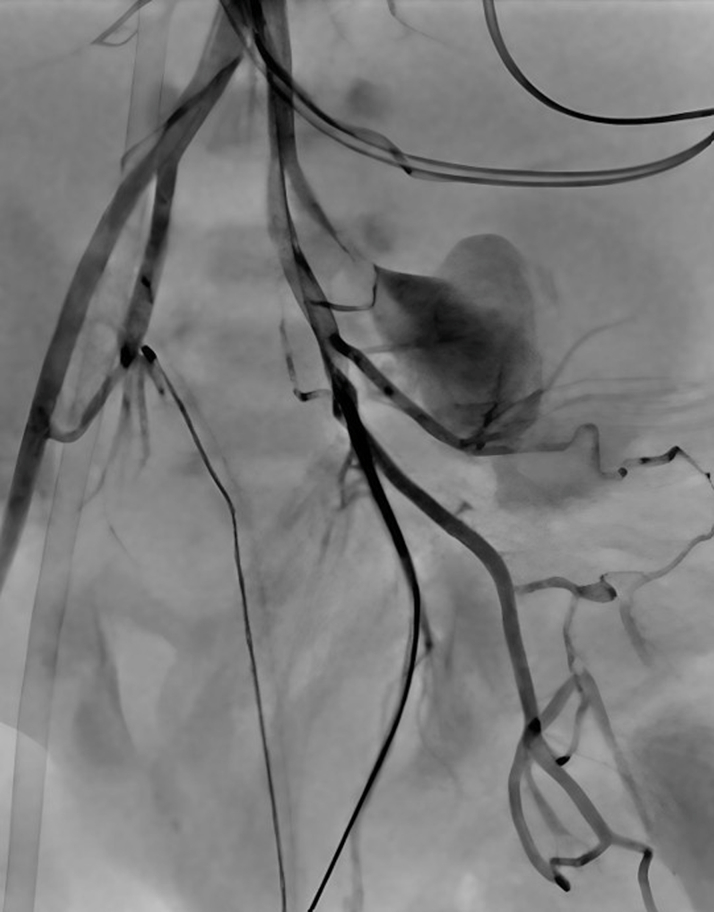
Left External Iliac Artery Pseudoaneurysm Angiographic still image demonstrating a pseudoaneurysm arising from the left external iliac artery. A contrast blush is visible, indicating extravasation into the surrounding tissue.

**Figure 4:**
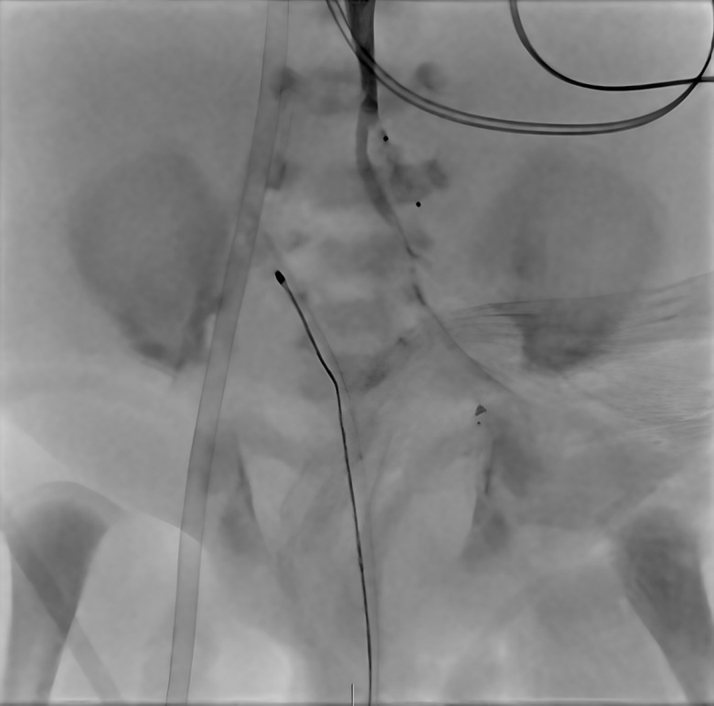
Pseudoaneurysm After Konar-A Vascular Plug Closure Follow-up angiogram after Konar-A vascular plug closure demonstrates no further contrast extravasation and preservation of distal femoral arterial branches.

Within 24 hours of transfer, the patient became increasingly hypoxemic, requiring maximal ionotropic and ventilatory support to maintain saturations >70%. Given concerns for restricted pulmonary flow, a restrictive atrial septum, and an enlarging pseudoaneurysm compromising perfusion to the distal left lower extremity, she underwent urgent catheterization on DOL 17 via right carotid access. Balloon atrial septostomy was performed to alleviate restriction of systemic venous return, reduce systemic venous congestion, and improve left ventricular filling. Descending aortic angiography confirmed the pseudoaneurysm with collateral flow to the distal femoral artery (Figure 3). The aneurysm neck was occluded with a 4-mm Konar-A (KA) vascular plug (Figure 4). The MVP flow restrictor was removed using an ensnare catheter. Postprocedural angiography demonstrated balanced ductal flow and improved hemodynamics.

## Outcome and Follow-Up

The patient tolerated the interventions without immediate complication. The pseudoaneurysm remained stable during her hospitalization; however, progressive left ventricular ischemia necessitated placement of a single ventricle assist device on DOL 37, improving function and perfusion. She was listed for heart transplant due to RV-dependent coronary circulation but was delisted after clinical decline and persistent oxygen needs. After discussion of medical futility with her family, her care was withdrawn, and she died on DOL 135.

## Discussion

The clinical course of our patient underscores the complexity of managing neonates with PA/IVS and RV-dependent coronary circulation, which involves a fine balance between pulmonary over- and undercirculation. Although these challenges have been well described in the literature, the novelty of our report lies in the dual use of 2 off-label catheter-based devices—a modified MVP for ductal flow regulation and a KA plug for pseudoaneurysm closure—in a single fragile neonate.

The Medtronic MVP, typically used for vascular embolization or PDA occlusion, has recently gained attention for its repurposing as a ductal flow restrictor in select single-ventricle lesions. In our case, dual fenestrations were created to tailor flow through an existing ductal stent. Although initially mitigating pulmonary overcirculation, subsequent undercirculation and hypoxemia highlighted the narrow hemodynamic window in PA/IVS and the need for continuous hemodynamic reassessment. Our experience builds on emerging literature that has demonstrated the feasibility and adaptability of fenestrated or manually modified MVPs for flow restriction to achieve balanced Q_p_/Q_s_ ratios in neonates with single-ventricle physiology, potentially offering an alternative to surgical pulmonary banding.^1^, ^2^, ^3^

The KA plug, designed for atrial or ductal occlusion, offered a minimally invasive solution for the iatrogenic left external iliac pseudoaneurysm. Although iatrogenic pseudoaneurysm formation in neonates is rare, similar cases have been described in the literature, with most developing after femoral or brachial arterial access, and treatment approaches range from surgical repair to percutaneous closure or conservative management and observation.^4^, ^5^, ^6^ Comparable percutaneous techniques for arterial pseudoaneurysm closure in older pediatric and adult populations have been reported, emphasizing device-based embolization as a safe, minimally invasive option when anatomy allows.^7^^,^^8^ To our knowledge, this represents the first use of a KA plug for neonatal iliac pseudoaneurysm repair in a patient with PA/IVS and RV-dependent coronary circulation. The device's flexible waist and controlled release provided precise deployment, avoiding open repair risks in this hemodynamically tenuous neonate. Our successful adaptation to this method for the neonatal population reinforces the potential for extending catheter-based strategies to increasingly younger and smaller patients. Particularly for small neonates requiring interventions such as the ones described in our case, our team advocates the use of carotid or axillary access because femoral access carries higher risks of vascular complications.

This case highlights how creative transcatheter adaptations can bridge gaps when traditional approaches are unfeasible. Although our patient's outcome was ultimately fatal, these interventions temporarily stabilized her hemodynamics and extended survival, demonstrating proof of concept for future similar patients.

Despite encouraging procedural results, long-term data on modified MVPs and vascular plugs in neonates are lacking. Future prospective multicenter registries are warranted to assess survival, complication rates, and ideal patient selection. Additionally, although off-label device use can be lifesaving, it requires risk-benefit assessment, rigorous institutional review, and multidisciplinary collaboration.

## Conclusions

This case underscores the extreme fragility of coronary and pulmonary hemodynamics in neonates with PA/IVS and RV-dependent coronary circulation. When conventional surgical options are unsuitable, creative catheter-based strategies—such as fenestrated MVPs for flow modulation and vascular plugs for pseudoaneurysm repair—offer lifesaving, minimally invasive alternatives. Early recognition of at-risk anatomy and timely consideration for cardiac transplantation remain critical to survival in these high-risk neonates.

## Funding Support and Author Disclosures

The authors have reported that they have no relationships relevant to the contents of this paper to disclose.

**Take-Home Messages**

• In PA/IVS with RV-dependent coronary circulation, balancing coronary and pulmonary flows is critical and often precarious.
• Creative use of transcatheter tools, including off-label applications, may provide lifesaving alternatives when surgical options are limited.

## Appendix

Video 1 Fluoroscopic Anteroposterior View Post-MVP Placement Fluoroscopic anteroposterior angiographic view after Medtronic microvascular plug (MVP) placement within the ductal stent, demonstrating regulation of ductal flow and visualization of the branch pulmonary arteries.

Video 2 Fluoroscopic Lateral View Post-MVP Placement Fluoroscopic lateral angiographic view after Medtronic microvascular plug (MVP) placement within the ductal stent, demonstrating regulation of ductal flow and visualization of the branch pulmonary arteries.
